# Competitive ability depends on mating system and ploidy level across *Capsella* species

**DOI:** 10.1093/aob/mcac044

**Published:** 2022-03-25

**Authors:** Marion Orsucci, Xuyue Yang, Theofilos Vanikiotis, Maria Guerrina, Tianlin Duan, Martin Lascoux, Sylvain Glémin

**Affiliations:** 1 Department of Ecology and Genetics, Evolutionary Biology Centre and Science for Life Laboratory, Uppsala University, 75236 Uppsala, Sweden; 2 Department of Ecology, Environment and Plant Sciences, Stockholm University, Stockholm, Sweden; 3 Department of Biological Applications & Technology, University of Ioannina, Leof. S. Niarchou GR-451 10, Ioannina, Greece; 4 UMR CNRS 6553 ECOBIO, Campus Beaulieu, bât 14a, CS 74205, 35042 Rennes, France; 5 Department of Plant Biology, Swedish University of Agricultural Sciences, Uppsala, Sweden

**Keywords:** Mating system, ploidy, life history traits, environmental disturbance

## Abstract

**Background and Aims:**

Self-fertilization is often associated with ecological traits corresponding to the ruderal strategy, and selfers are expected to be less competitive than outcrossers, either because of a colonization/competition trade-off or because of the deleterious genetic effects of selfing. Range expansion could reduce further competitive ability while polyploidy could mitigate the effects of selfing. If pollinators are not limited, individual fitness is thus expected to be higher in outcrossers than in selfers and, within selfers, in polyploids than in diploids. Although often proposed in the botanical literature and also suggested by meta-analyses, these predictions have not been directly tested yet.

**Methods:**

In order to compare fitness and the competitive ability of four *Capsella* species with a different mating system and ploidy level, we combined two complementary experiments. First, we carried out an experiment outdoors in north-west Greece, i.e. within the range of the obligate outcrossing species, *C. grandiflora*, where several life history traits were measured under two different disturbance treatments, weeded plots vs. unweeded plots. To better control competition and to remove potential effects of local adaptation of the outcrosser, we also performed a similar competition experiment but under growth chamber conditions.

**Key Results:**

In the outdoor experiment, disturbance of the environment did not affect the phenotype in any of the four species. For most traits, the obligate outcrossing species performed better than all selfing species. In contrast, polyploids did not survive or reproduce better than diploids. Under controlled conditions, as in the field experiment, the outcrosser had a higher fitness than selfing species and was less affected by competition. Finally, contrary to the outdoor experiment where the two behaved identically, polyploid selfers were less affected by competition than diploid selfes.

**Conclusions:**

In the *Capsella* genus, selfing induces lower fitness than outcrossing and can also reduce competitive ability. The effect of polyploidy is, however, unclear. These results highlight the possible roles of ecological context in the evolution of selfing species.

## INTRODUCTION

Mating system shift and polyploidization events are two major evolutionary transitions in plant evolution that can eventually lead to speciation by promoting both reproductive isolation and ecological divergence (Otto and Whitton, 2000; Wright *et al.*, 2013).

Mating system shift from outcrossing to self-fertilization occurred repeatedly during plant evolution, most probably since it provides reproductive assurance and because of the transmission advantage of selfing mutations (Stebbins, 1974; Barrett, 2002). In addition to well-documented changes in floral morphology (selfing syndrome), outcrossing and selfing sister species may also diverge ecologically: selfing plants no longer depend on pollinators and therefore can be more frequently found in disturbed or patchy habitats and be better at colonizing new environments (‘Baker’s law’; Pannell, 2015). Selfing is also often associated with invasiveness (Van Kleunen *et al.*, 2008) and selfing species tend to have larger geographic ranges than their outcrossing relatives (Van Kleunen and Johnson, 2007; Randle *et al.*, 2009; Grossenbacher *et al.*, 2015). In contrast, outcrossers could be better competitors (Munoz *et al.*, 2016) and would not suffer from the negative genetic effects of inbreeding (lack of genetic diversity, accumulation of deleterious mutations; Stebbins, 1957; Wright *et al.*, 2008; Igic and Busch, 2013; Glémin *et al.*, 2019). They would also suffer less from the costs associated with demographic expansion during colonization, the so-called ‘expansion load’ (Peischl *et al.*, 2013; Peischl and Excoffier, 2015). Alternatively, selection for better colonizing ability in a selfer could be at the cost of lower competitive ability (Bossdorf *et al.*, 2004; Burton *et al.*, 2010). Consequently, in a given environment where pollinators are not limited, outcrossing species should have a higher fitness than their self-fertilizing relatives, and this difference should be enhanced under a more competitive environment.

Polyploid self-fertilizing species make this simple model somewhat more complex. Polyploidy is often associated with selfing (Barringer, 2007; Robertson *et al.*, 2011) and could favour the evolution of selfing by breaking down self-incompatibility systems (Mable, 2004) and reducing the impact of inbreeding depression by masking the effect of recessive deleterious alleles, thanks to the retention of multiple gene copies (Ronfort, 1997; Otto and Whitton, 2000). Polyploidy can initially challenge proper meiosis and reproduction (Bomblies *et al.*, 2016); however, if these problems are overcome, polyploids can have greater fitness than their diploid relatives (an idea dating back to Shull, 1929) and acquire new beneficial traits (e.g. salt resistance). Detailed fitness comparisons between polyploids and their diploid relatives have increasingly supported these predictions (e.g. Wei *et al.*, 2019; Forrester *et al.*, 2020) but sometimes also showed the opposite pattern (e.g. Siopa *et al.*, 2020). It is thus relevant to also consider a possible polyploidy effect in relation to the evolution of selfing.

According to the rationale presented above, we predict that (1) transition from outcrossing to selfing should lead to reduced fitness, especially under competitive conditions; (2) this effect should be less pronounced in polyploid than in diploid selfers; and (3) within species, competitive ability should decline during range expansion.

The *Capsella* genus (Brassicaceae) has some distinct advantages to address such questions. While it is a small genus with only four species, it combines species with different ploidy levels and mating systems. Indeed, three species are diploid and one is tetraploid. *Capsella grandiflora* (Fauché & Chaub.) Boiss is a diploid and obligate outcrosser (2*x* = 16), with a sporophytic self-incompatibility system, which is geographically restricted to northern Greece and Albania. *Capsella rubella* Reut. and *C. orientalis* Klokov are both predominant selfers (2*x* = 16). *Capsella rubella* derived from *C. grandiflora* approx. 25 000–40 000 years ago (Foxe *et al.*, 2009; Guo *et al.*, 2009) and its origin seems to be concomitant with the breakdown of the self-incompatibility system (Guo *et al.*, 2009). *Capsella rubella* grows around the Mediterranean and as far north as Belgium in western Europe. The main distribution area of *C. orientalis* extends from central Ukraine to north-western China and western Mongolia (Hurka *et al.*, 2012). *Capsella bursa-pastoris* (L.) Medik. (Brassicaceae) is an allotetraploid and predominantly self-fertilizing species (4*x* = 32) with disomic inheritance, arising from the hybridization between the ancestors of *C. grandiflora* and *C. orientalis* (approx. 100 000–300 000 years; Douglas *et al.*, 2015). In contrast to its diploid relatives, it has an almost worldwide distribution. At least three main, well-differentiated, genetic clusters can be defined, probably corresponding to colonization events from the Middle East (the probable centre of origin) to Europe and then to Eastern Asia. Across its expansion range, genetic diversity decreases and deleterious mutations accumulate from the Middle East to Eastern Asia (Cornille *et al.*, 2016; Kryvokhyzha *et al.*, 2016), in agreement with population genetic predictions on range expansion dynamics (Excoffier *et al*., 2009; Peischl *et al*., 2013; Peischl and Excoffier, 2015).

A previous study has investigated differences in competitive abilities in the genus *Capsella* according to ploidy level and mating system (Petrone Mendoza *et al.*, 2018), showing that selfing species were more sensitive to competition than the outcrossing species but that polyploidization of *C. bursa-pastoris* could counteract the negative effect of selfing. However, this first study had three main limitations: (1) *C. orientalis* was missing while it offers an independent test of the effect of selfing; (2) the sampling scheme was not representative of the whole species range as it focused on Greek populations where the three species (*C. grandiflora*, *C. bursa-pastoris* and *C. rubella*) are sympatric so the predictions of the effect of range expansion associated with selfing were not tested; and finally (3) the study was carried out in growth chambers where the outcrossing species could not be pollinated and did not produce seeds. Thus, absolute fitness between selfers and outcrossers could not be directly compared and the analysis only focused on the relative effect of competition between species. In addition, the overproduction of flowers in *C. grandiflora* in the absence of pollinator could have altered the estimation of the effect of competition.

To circumvent these problems, we extended the sampling scheme to the four species and to their whole species range, especially for *C. bursa-pastoris* that shows the strongest population structure and signature of range expansion. We also carried out two complementary experiments. First, we installed a common garden experiment within the natural range of *C. grandiflora*, *C. rubella* and *C. bursa-pastoris*, in northern Greece. To evaluate the sensitivity of *Capsella* species to competition, we grew the plants under two different environmental treatments: ‘weeded’ (i.e. other plant species are constantly removed) and ‘unweeded’ (i.e. other plant species can grow around the focal *Capsella* plant). Second, we performed a new controlled competition experiment, whose design is similar to that of Petrone Mendoza *et al.* (2018) to allow for a better control of competitive conditions than in the natural environment and to compare the two conditions for all species across their species range.

## MATERIALS AND METHODS

### Field competition experiment

#### Study material.

We used ten accessions of *C. rubella* from seven populations, six accessions of *C. orientalis* from four populations, ten genotypes from seven different populations of *C. grandiflora*, the outcrossing species, and 18 accessions of *C. bursa-pastoris* from 18 populations, including five accessions from the European genetic cluster (EUR), four from the Middle-Eastern cluster (ME), six from the Asian genetic cluster (ASI) and finally three from Central Asia (CASI; Table 1; for details of the accessions, see Supplementary data Table S1).

**Table 1.** Experimental details for the two experiments used in the present study and flowering percentage per species and for each geographical origin of *C. bursa-pastoris*

Experiment	Species		No. of accessions used	No. of individuals transplanted	Flowering percentage
Semi-natural conditions	*C. grandiflora*		10	80	98.75
	*C. orientalis*		6	48	89.58
	*C. rubella*		10	80	96.25
	*C. bursa-pastoris*	Total	18	288	98.26
		ASI	6	96	100
		CASI	4	64	98.44
		EUR	4	64	93.75
		ME	4	64	100
Controlled conditions	*C. grandiflora*		13	104	87.5
	*C. orientalis*		19	152	100
	*C. rubella*		33	264	78.4
	*C. bursa-pastoris*	Total	49	152	90.1
		ASI	18	144	82.6
		CASI	5	32	87.5
		EUR	17	136	95.6
		ME	9	72	100

ASI, Asian accession; CASI, Central Asia accession; EUR, European accession; and ME, Middle-Eastern accession.

#### Experimental design.

 The experiment was carried out on the campus of the University of Ioannina (Greece) (39°37.090′N and 20°50.806′E), which is located within the natural range of *C. grandiflora* and where *C. rubella* and *C. bursa-pastoris* are also naturally occurring. Differences in ability to withstand competition between *Capsella* species were tested under two different treatments: (1) weeded, where the vegetation was removed once or twice a week around the *Capsella* focal plant; and (2) unweeded, without removal of the vegetation.

First, at least 50 seeds per accession were sown into plastic pots (9 × 6.5 cm) containing standard soil (Klasmann Traysubstrat) in January 2018. Pots were stratified for 7 d by keeping them in the dark at 4 °C and then moved into a growth chamber with 16/8 h light/darkness cycles at 22 °C to allow germination. Germination of the earliest accessions started approx. 10 d later (Supplementary data Table S2). Seedlings that presented two pairs of well-developed first true leaves were transplanted into single square pots (8 × 8 cm). In each pot, two seedlings were planted on opposite corners. At the beginning of March 2018, all pots were moved to an experimental garden for a phase of acclimation to external conditions.

Two weeks before transplantation in the experimental plots, vegetation and larger stones were removed from the experimental field, thereby homogenizing the topsoil. The experimental field was then covered with anti-weed tissue, in which 10 × 10 cm holes separated by 20 cm were made to create experimental plots. To facilitate transplantation, vegetation was removed in all plots just before performing the transplants. Then, vegetation was removed once to twice a week in plots corresponding to the weeded treatment, while plots corresponding to the unweeded treatment were left untouched.

From 7 to 14 April, a total of 496 plants were distributed randomly (sample function, R version 3.6.3; R Development Core Team, 2020) over the two different treatments which were organized in four experimental groups. The 496 plants corresponded to eight replicates of 18 accessions of *C. bursa-pastoris*, four replicates of ten accessions of *C. grandiflora*, four replicates of ten accessions of *C. rubella* and four replicates of six accessions of *C. orientalis*. In addition, in each treatment, 20 plots (also distributed randomly) were left empty in order to monitor spontaneous vegetation recolonization.

The performance of the four *Capsella* species under the two different treatments (weeded vs. unweeded) was monitored by recording for each plant: (1) vegetative traits such as rosette diameter (measured on the day of transplantation) and number of floral stems per plant (measured on dead plants); (2) phenological traits such as flowering start (i.e. number of days between plant transplantation and the first flower) and life span (i.e. number of days between plant transplantation and death); and (3) main fitness components, i.e. fertility measured as the number of fruits, which is a proxy of the number of offspring, and viability which was quantified by seed germination rate. In addition, we computed a fitness index (*W*_index_), which is an integrative measure of overall performance and is defined as the product of the number of fruits and germination rate. The combination of these two components provides a better estimate of the overall fitness of each species (and genetic cluster within *C. bursa-pastoris* species). In addition to these traits, damage (e.g. cut stems or an unhealthy plant) on the plants, which could be caused by phytophagous insects (ants or aphids) or water stress, were recorded and summarized by a single variable called damage (see below).

For all four species, seeds were collected on each flowering plant to quantify the germination rate. One year after the common garden experiment was over, 50 seeds were randomly sampled on each plant and were sown directly in soil of square pots (4 × 4 cm) that were randomized. After 10 d of stratification (24 h dark, 4 °C), the pots were moved into growth chambers (12:12 h light:dark, 22 °C). The number of seedlings after 21 d was recorded and the germination rate was calculated as the number of seedlings over the number of seeds sown.

#### Statistical analysis.

 All statistical analyses were performed using the R software (R version 3.6.3; R Development Core Team, 2020). First, in order to evaluate the contributions of each trait to the variation among species, principal component analyses (PCAs) were performed on all traits with the function dudi.pca (ade4 package; Dray and Dufour, 2007).

Then, for all traits used as the response variable (diameter of the rosette, life span, flowering start, number of fruits, germination rate and fitness index), linear models were used as follows: the fixed predictors were (1) the treatment (qualitative variable, two levels: weeded or unweeded); (2) the biological characteristics (qualitative variables, two levels for reproductive system, two levels for ploidy and four levels for species and genetic clusters within *C. bursa-pastoris*; see above for more details); and (3) the interaction between the two predictors. We added one co-variable, which measures damage (qualitative variable, two levels: damaged or not). In addition, a row effect (which is due to experimental constraints) and an accession effect were included as random effects in each model.

We analysed the rosette size (quantitative variable) and the flowering start (LOG10 normalization) with a linear model, assuming a Gaussian error distribution (lmer function, stats package, R version 3.6.3; R Development Core Team, 2020). Life span, number of fruits, fitness index and germination rate were analysed using a generalized linear mixed model, assuming a negative binomial distribution, except for the last one where we assumed a binomial distribution (glmer.nb and glmer functions, respectively, lme4 package, Bates *et al.*, 2015).

The significance of the fixed effects was tested with deviance analyses using the anova function of the car package in R (Fox and Weisberg, 2019). Interactions were tested first [type III analysis of variance (ANOVA)], then, if the interaction was not significant, a model without interaction term was run to test only the main fixed effects (type II ANOVA). The significance of difference between factor levels was tested by a post-hoc procedure allowing multiple comparisons (glht function, multcomp package, Hothorn *et al.*, 2008).

### Controlled competition experiment

#### Study material.

 Fifteen to 67 accessions from each *Capsella* species sampled over their respective distribution range were used for interspecific comparisons (Supplementary data Table S1). To account for the worldwide distribution of *Cbp,* we used 67 accessions, including 31 from Asia, 22 from Europe, nine from the Middle East and five from Central Asia (Supplementary data Table S1). While the same species and the same genetic clusters were represented in the two experiments, there was otherwise limited overlap between the two experiments at the accession or population levels (see Supplementary data Table S1). Petrone Mendoza *et al.* (2018) showed that the difference in sensitivity to competition was only weakly affected by which species was used as the competitor, either one of the *Capsella* species or *Matricaria chamomilla*, an annual Asteraceae species, which co-occurs with *Capsella* species in Greek populations (pers. obs.). To avoid mixing intra- and interspecific competition by using one of the *Capsella* species as competitor, we only used *M. chamomilla* in this experiment. As in Petrone Mendoza *et al.* (2018), we used commercial seeds of *M. chamomilla*, in order to ensure good germination and homogeneity among plants.

#### Experimental design.

Each accession was tested without and with four competitors in a complete random block design. More specifically, one focal *Capsella* individual was sown in the middle of an 11 × 11 × 11 cm pot, either alone or surrounded by four *M. chamomilla* competitors. Each combination accession × treatment was replicated four times.

First, for each accession, 30 seeds were sown in agar plates and stratified, i.e. put in darkness at 4 °C for 6 d. Then, agar plates were moved to a growth chamber (22 °C constant, 16:8 h light:darkness, under a light intensity of 130 μmol m^–2^ s^–1^) for germination. After 5 d, germinated competitors were transferred to pots filled with standard culture soil and the pots were placed randomly in two growth chambers. Each growth chamber contained two blocks, and light and temperature conditions were the same as for germination. After recording the germination rates of each accession, focal species individuals were planted 5 d after their competitors in order to ensure a strong enough competition against the *Capsella* species. Accessions with fewer than eight germinated seeds were not used.

On focal individuals, we first measured the rosette growth rate: two perpendicular diameters of the rosette were measured first at 3 weeks after sowing and then again 1 week later. The product of these two diameters was used as a proxy for rosette surface at time *t*1 and *t*2, respectively (S1 and S2). We then calculated the rosette growth rate as the relative difference of the two rosette surfaces: (S2 – S1)/S1; and the total number of flowers – the total number of flowers was counted when plants started to senesce. We used the number of flowers to allow comparison between the four species because we could not use the number of fruits as in the field experiment since *C. grandiflora* s*et al*most no fruit due to the absence of pollinators in the growth chambers.

#### Statistical analysis.

 For a given trait, the fixed predictors were (1) the species (qualitative variable, four levels) or the genetic clusters for *C. bursa-pastoris* (qualitative variable, three levels); (2) the treatment (qualitative variable, two levels); and (3) all pairwise interactions. The accession and block variables were included as random effects in each model.

We analysed the rosette surface and growth rate (quantitative variable) using linear mixed models (lmer function, lme4 package, Bates *et al.*, 2015). The number of flowers was analysed using a generalized linear mixed model, assuming a negative binomial distribution, except for the last measurement where we assumed a binomial distribution (glmer.nb and glmer functions, respectively, lme4 package, Bates *et al.*, 2015). The distribution of the number of flowers was bimodal with a mode at 0 and another at around 1000. It was therefore analysed in two steps. First, we analysed the proportion of flowering plants (note that dead plants were included in the non-flowering category) with a binomial model (glmer function, lme4 package, Bates *et al.*, 2015). Then we excluded plants that did not flower and analysed the number of flowers using a negative binomial distribution (glmmadmb function, glmmADMB package, Fournier *et al*., 2012; Skaug *et al*., 2013).

To determine whether the effect of competition differed among species, we specifically focused on the ‘species × treatment’ interaction term (or the ‘area × treatment’ interaction for the comparison within *C. bursa-pastoris*). To get a more direct estimate and more intuitive interpretation of the result, we computed a competition index, as defined in Petrone Mendoza *et al.* (2018):


$$\begin{array}{l} I_{c}=\frac{W_{competition}}{W_{alone}} \end{array}$$

where $W_{alone}$ and $W_{competition}$ are the least-square mean of a trait without and with competition, respectively. Mean effects were estimated with the lsmean function of the lmerTest package (Kuznetsova *et al.*, 2017). The lower the *I*_c_, the more sensitive to competition the species or the geographic group is.

The significance of the fixed effects was tested with deviance analyses using an ANOVA (anova function, car package, Fox and Weisberg, 2019). For linear mixed models, we used the anova function of the standard R distribution. For generalized linear mixed models, we performed analyses of deviance employing the anova function of the car package using the type III ANOVA option (Fox and Weisberg, 2019). For linear mixed models, random effects were tested by likelihood ratio tests using the rand function of the lmerTest package (Kuznetsova *et al.*, 2017). For generalized linear mixed models, we also ran the equivalent models without random effects and compared the likelihood of the models with the anova function.

## RESULTS

### Field competition experiment

#### Strong differences between the selfing and outcrossing species.

**Fig. 1.**
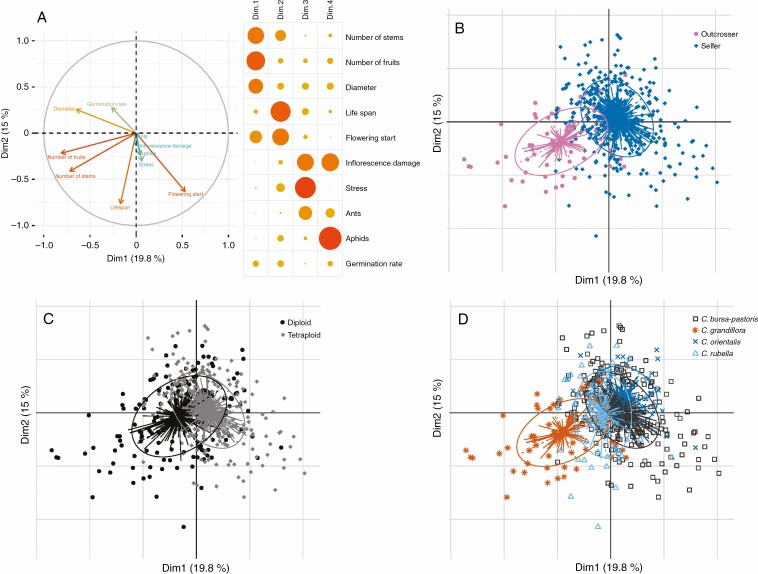
Principal component analysis (PCA) based on life history traits measured in *Capsella* spp. (A) Correlation circle (left panel) showing the relative contribution of each variable to the two first principal components explaining 35 % of the total observed variance; the right panel shows the relative contribution of each variable to the four first principal components (the darker and the larger the discs, the higher the contribution). (B–D) PCA with all individuals using different grouping to highlight differences relative to mating system (B), ploidy level (C) and species (D).

**Fig. 2.**
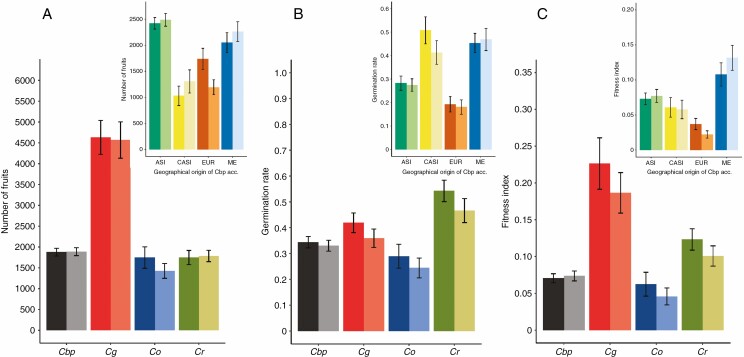
Life history traits and fitness index for *Capsella* species (main figure) and between genetic clusters of *C. bursa-pastoris* (top right panels). The mean number of fruits (A), the mean germination rate (B) and the mean fitness index (C) which is scaled to vary between 0 and 1 $\left(F_{INDEX}=\frac{GR\;\times \;N_{F}}{\max(GR\;\times \;N_{F})}\;\right)$ where GR is the germination rate and N_F_ is the number of fruits, meaning that the higher the index, the higher the relative fitness. The three traits are represented for *C. bursa-pastoris* (*Cbp*), *C. grandiflora* (*Cg*), *C. orientalis* (*Co*) and *C. rubella* (*Cr*) or genetic clusters of *C. bursa pastoris* (ASI, CASI, EUR and ME) in weeded (dark shading) and unweeded (light shading) treatments. The corresponding standard errors are indicated by bars.

 The two first principal components of the PCA including all traits explained 35 % of the total observed variance (Fig. 1A), with the first component discriminating the two reproductive systems (Fig. 1B) and, to a lesser extent, the two ploidy levels (diploid vs. tetraploid, Fig. 1C). Most traits contribute to these two first components except the highly correlated traits ants, aphids, stress and inflorescence damage (Fig. 1A): we then merged them into a unique variable called damage and used it as a covariable in subsequent analyses. Except for flowering start, the outcrossing species, *C. grandiflora*, differed from selfing species for all other traits. First, the rosette diameter was significantly larger in outcrossing individuals (approx. 12.3 ± 2.6 cm) than in selfing plants (10.6 ± 3.2 cm; Table 2; Supplementary data Table S3). Second, the outcrossers produced about 2.5 times more fruits than selfers (out. = 4668, self. = 1884; Supplementary data Table S3; Fig. 1A). This can be related to the extended life span of the outcrossers (out. = 130, self. = 118 d; d.f. = 474, *z* = –6.92, *P* < 0.001; Table 2; Supplementary data Table S3; Fig. S1). However, flowering start did not differ significantly between the two groups (out. = 16, self. = 17 d d.f. = 474, *t* = 0.219, *P* = 0.83; Table 2). Finally, germination rate was also on average higher among outcrossing accessions than among those which were self-fertilizing (out. = 0.39, self. = 0.35; Fig. 1B; Supplementary data Table S3). The fitness index magnified the differences between the four species (Fig. 2C). When the number of fruits and germination rate are jointly considered, selfing species have a lower fitness than *C. grandiflora* (Fig. 2C; Supplementary data Table S3).

**Table 2.** Mean values for traits indirectly associated with fitness in the outdoor experiment in north-west Greece

			Rosette size (cm)			Flowering start (d)			Lifes pan (d)		
Species		*n*	Mean	s.e.		Mean	s.e.		Mean	s.e.	
*Cbp*		283	10.36	0.20	a	17	0.42	a	118	0.83	a
	ASI	96	9.06	0.31	a	16	0.43	ab	116	1.36	a
	CASI	64	10.12	0.34	a	23	0.99	c	123	1.65	b
	EUR	64	10.57	0.54	a	19	0.92	b	117	1.94	ab
	ME	64	11.98	0.40	a	12	0.56	a	115	1.71	a
*Cg*		77	12.32	0.30	b	16	0.46	a	130	2.19	b
*Co*		43	10.36	0.40	a	14	0.87	b	113	2.00	a
*Cr*		77	11.92	0.28	b	17	0.71	a	122	1.98	b

The means for the four *Capsella* species are in black and the means per geographical origin within *C. bursa-pastoris* are in grey

*Cbp*, *Capsella bursa-pastoris*; *Cg*, *C. grandiflora*; *Co*, *C. orientalis*; *Cr*, *C. rubella*; ASI, Asian accessions; CASI, central Asian accessions; EUR, European accessions; ME, Middle-Eastern accessions. Different letters for each trait indicate significant differences. Those in grey are related to the comparison among *Cbp* accessions.

Unexpectedly, no differences between weeded and unweeded treatments were observed for most of the response variables (Supplementary data Fig. S2, except for germination rate, Supplementary data Fig. S1; Table S3) so we could not test whether the selfing species were more sensitive to competition than the outcrossing *C. grandiflora*.

#### No or weak difference between diploid and polyploid selfers.

Because of the strong effect of the mating system, we limited the analysis of the effect of ploidy to the three selfing species. Ploidy had a weak and, generally non-significant, effect on most traits (Supplementary data Table S3), except for rosette size that was slightly and significantly smaller in *C. bursa-pastoris* (diameter = 10.2 ± 3.4 cm) than in *C. rubella and C. orientalis* (diameter = 11.1 ± 3.1 cm, *P* < 0.001; Table 2; Supplementary data Table S3). In contrast, the fitness index differed significantly among species but not in relation to ploidy levels: *C. rubella* performed better globally than *C. bursa-pastoris* (d.f. = 444, *z* = 3.364, *P *< 0.01; Fig. 2C) and *C. orientalis* (d.f. = 444, *z* = 3.138, *P *< 0.01; Fig. 2C), the last two species being similar (d.f. = 444, *z* = –1.322, *P *= 0.53; Fig. 2C). However, Middle-Eastern accessions within *C. bursa-pastoris*, which originate from regions with similar conditions to those of our site of experimentation, do not differ from *C. rubella* (d.f. = 127, *z* = –0.51, *P *= 0.61; Fig. 2C).

#### *Variation within* C. bursa-pastroris.

Some of the four *C. bursa-pastoris* genetic clusters differed significantly (Supplementary data Fig. S3), though differences were not consistent across the different traits. The ranking of the fitness index also changed among the four *C. bursa-pastoris* genetic clusters: while Asian accessions produced a significantly higher number of fruits than plants from the other three genetic clusters, they had a lower overall performance than Middle-Eastern accessions when the germination rate was also included (d.f. = 265, *z* = 3.408, *P *< 0.01; Fig. 2).

### Controlled competition experiment

#### Data overview.

 After having discarded accessions with too few seeds, the final dataset contained 13 accessions of *C. grandiflora*, 33 of *C. rubella*, 19 of *C. orientalis* and 49 of *C. bursa-pastoris*. The 49 *C. bursa-pastoris* accessions were distributed over the four geographic regions as follows: nine from the Middle East, 17 from Europe, five from Central Asia and 18 from China (Table 1). Survival rates were very high (>90 %) for all species, and most plants flowered.

**Fig. 3.**
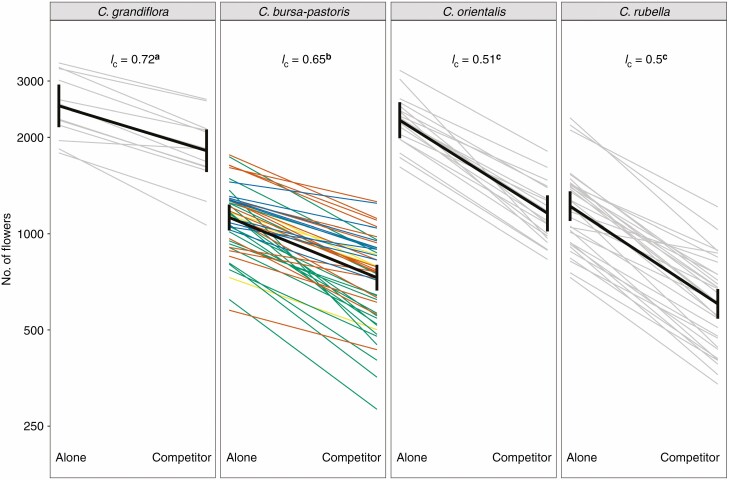
Flower number without and with competitors in the four species. Each thin line corresponds to one accession (averaged over the four blocks) and the black lines join the least-square mean estimates (with confidence intervals). For *C. bursa-pastoris*, the colour of each line corresponds to the genetic clusters: ASI in green, CASI in yellow, EUR in orange and ME in blue. *I*_c_: competition index. *I*_c_ with different letters corresponds to significant treatment × species interactions (see Supplementary data Table S4).

In contrast to the field experiment, a strong competition effect (Fig. 3; Supplementary data Fig. S4) was detected under the controlled environment. The higher level of competition observed under the controlled environment is probably caused by the very limited resources available in plastic pots. Otherwise, and once again, the reproductive system had the strongest effect, with *C. grandiflora* outperforming all its selfing relatives (Fig. 3).

#### *Differences between* Capsella *species.*

Rosette surfaces at the two time points, but not growth rate, were significantly and negatively affected by competition (Supplementary data Fig. S4; Table S4), reducing the rosette surface by around 5 %. There is no species effect (all *P* > 0.1; Supplementary data Table S4) on these vegetative traits, which were affected similarly by the treatment effect (*P* > 0.1 for species × treatment interaction; Supplementary data Table S4). In contrast, strong competition and species effects were detected on flower number (*P* < 0.001; Supplementary data Table S4). In addition, the species were not equally affected by the competition treatment (*P* < 0.001 for species × treatment interaction; Supplementary data Table S4): the two diploid selfing species, *C. orientalis* and *C. rubella* [*I*_c_ = 0.52; likelihood ratio test (LRT) *Co*–*Cr*: χ ^2^ = 0.55, *P* = 0.46] were more affected by competition than *C. grandiflora*, which was the least affected (*I*_c_ = 0.74; LRT *Cg*–*Cr/Co*: χ ^2^ = 53.53/40.85, *P* < 0.001). The tetraploid *C. bursa-pastoris* was intermediate with *I*_c_ = 0.65 (LRT *Cbp*–*Cr/Co*: χ ^2^ = 54.57/35.37, *P* < 0.001; LRT *Cbp*–*Cg*: χ ^2^ = 5.16, *P* = 0.03; Fig. 3).

#### *Differences due to the geographical origin of* Capsella bursa-pastoris.

 A negative effect of competition on rosette surfaces and growth rate was detected (*P *< 0.05; Supplementary data Table S4). In addition, a significant area × treatment effect was observed for the second measure of rosette surface (Supplementary data Fig. S4; Table S4). The number of flowers was significantly affected by competition but the strength of the effect varied across accessions (Fig. 3; Supplementary data Table S4; area × treatment interaction, *P* < 0.001). Asian accessions were the most sensitive to competition (*I*_c_ = 0.54) and Middle-Eastern accessions the least (*I*_c_ = 0.81), Central Asia and Europe being intermediate with 0.71 and 0.69, respectively (Fig. 3; LRT AS–ME/EUR/CASI: χ ^2^ = 34.20/22.83/8.24, all *P* < 0.01; LRT ME–EUR/CASI: χ ^2^ = 3.20/1.31, all *P* > 0.1).

## DISCUSSION

In the present study, we combined the results of two complementary competition experiments in the *Capsella* genus. Under controlled conditions, and as previously observed in Petrone Mendoza *et al.* (2018) and Orsucci *et al.* (2020), competition had a strong effect on the number of fruits or flowers produced by the different species, but, unexpectedly, competition did not have any effect under field conditions, which were less controlled and resulted in a weaker than expected competition effect. However, in both experiments, *C. grandiflora* had a higher fitness than all three selfing species that did not differ significantly. So, in summary, mating system was the strongest factor affecting fitness components. Below, we discuss possible issues with, and implications of, these two experiments.

### *Fitness and competitive ability vary within and among* Capsella *species*

We found clear differences in mean fitness among the four species that matched our expectations since the outcrossing species had higher fitness than its self-fertilizing relatives in both field and growth chamber experiments (Figs 2 and 3). In the growth chamber experiment, *C. grandiflora* was not pollinated and did not produce fruits, which could lead to overestimating fitness in comparison with the selfing species since *C. grandiflora* could overproduce flowers compared with natural conditions when pollinators are not limiting. However, the number of fruits in the field was of the same order of magnitude for *C. orientalis* and even higher for *C. grandiflora*, *C. bursa-pastoris* and C*. rubella* than the number of flowers in the growth chamber. This suggests that the indirect measures of fitness under controlled conditions turned out to be rather good proxies of fitness differences among species under natural conditions.

In addition, in the controlled experiment, the outcrossing species had higher competitive ability than the three selfing species (Fig. 3). Because competition was measured as the difference in relative fitness, this result is robust to the above-mentioned issue of fitness overestimation in *C. grandiflora* (as in Petrone Mendoza *et al.*, 2018 and Orsucci *et al.*, 2020). However, this effect could not be confirmed under natural conditions because the environmental disturbances used in our field experiment did not lead to any fitness differences among species. Clearly, the natural environment (i.e. unweeded treatment) was much less competitive than we had expected, potentially because all plots were initially weeded to permit the transplantation of *Capsella* individuals, because they were maybe too close to each other so that root competition was the same for all plants and/or simply because resources in the field were not sufficiently limiting.

Evidence for the hypothesis that polyploidy could alleviate problems associated with self-fertilization was not clear. In some analyses, the tetraploid selfer performance was even worse than the performance of diploid selfers. For example, under controlled conditions, tetraploid plants produced fewer flowers than diploid plants (Fig. 3) and, in our field experiment, the fitness index of *C. bursa-pastoris* was even lower than that of *C. rubella* (Fig. 2). However, under controlled conditions, despite lower fitness on average, *C. bursa-pastoris* appeared less sensitive to competition than the two diploid selfers (Fig. 3). This could partly be explained by the geographic origin of the accessions. Individuals coming from plants that were sampled in Greece or around the Mediterranean area (i.e. all accessions of *C. rubella* and *C. grandiflora* and the accessions corresponding to the ME genetic cluster of *C. bursa-pastoris*) had a higher fitness index than those with a more distant geographical origin (i.e. all accessions of *C. orientalis* and the accessions corresponding to the ASI, CASI and EUR genetic cluster of *C. bursa-pastoris*). These genotypes could be maladapted to the environmental conditions at the site of the experiment, which could indirectly contribute to differentiation among selfing species and populations. Indeed, plant species often exhibit strong local adaptation (Savolainen *et al.*, 2007; Hereford, 2009). This could well be the case for *C. orientalis*, which exhibited a large number of flowers under controlled conditions (close to the flower production by *C. grandiflora*, Fig. 3) but was the species that produced the least fruits under field conditions in north-west Greece (Fig. 2A). In contrast, within *C. bursa-pastoris*, the ranking among genetic clusters does not differ across experiments, where ASI populations, corresponding to the expansion front of the species (Cornille *et al.*, 2016), performed the worst and were the least competitive. This is in line with their higher genetic load (Kryvokhyzha *et al.*, 2019) and with the limited evidence of local adaptation at large geographical scale where ASI populations performed the worst under three different environments, including their native environment, in China (Cornille *et al.*, in press).

### Extending the selfing syndrome to the whole life cycle?

When comparing selfing and outcrossing sister species, the focus is mainly on morphological and functional reproductive traits, collectively forming the selfing syndrome (Sicard and Lenhard, 2011). Our results revealed more extensive differences, including phenological, vegetative and fitness-linked traits. For both life cycle traits and fitness components, the strongest differences were observed between selfing and outcrossing species. In particular, *C. grandiflora* produced 2.5 times more flowers, had a reproductive period on average 14 d longer and a rosette size about 2 cm larger than the selfing species. These results cannot be simply explained by the phylogenetic relationships among the three selfing species that evolved independently. This suggests that evolution of selfing affects not only floral morphology but also many other traits, pointing to a more global and integrated selfing syndrome including both vegetative and ecological traits (see discussion in Petrone Mendoza *et al.*, 2018). This is in line with the time limitation hypothesis that posits that self-fertilizing annual plant species invest in rapid growth, but also little in developmental traits (decreasing flower and plant sizes), and have shorter bud development time and flower longevity in order to produce seeds rapidly, which compensates for their low chance of survival and low competitive ability (Aarssen, 2000; Snell and Aarssen, 2005). In addition, selfing species often exhibit a higher genetic load than their outcrossing relatives (Arunkumar *et al.*, 2015; Glémin *et al.*, 2019). This is the case in the *Capsella* genus (Kryvokhyzha *et al.*, 2019) and this could also contribute to lower their fitness.

### *High fitness and tiny range: the paradoxical case of* C. grandiflora

Selfing does not only bring disadvantages but also confers reproductive assurance over outcrossing and thereby facilitates the colonization of new territories. In the *Capsella* genus, all three selfing species have a much larger geographical range than their outcrossing relative, *C. grandiflora*, which has a very limited distribution range, being confined to a small geographical region in northern Greece and Albania. This limited geographical range of *C*. *grandiflora* is, at first glance, surprising considering that *C*. *grandiflora* seems to perform better than all its selfing relatives, and has a much higher genetic diversity (Kryvokhyzha *et al.*, 2019) and a better competitive ability than diploid selfers (Petrone Mendoza *et al.*, 2018; this study). Actually, the factors limiting its spread are still unclear, but it does not seem to be due to a dependence on specific pollinators (pers. obs. and pers. comm.). While selfing species have larger geographic ranges than their outcrossing close relatives, due primarily to more limited dispersal caused by mate limitation in outcrossers outside of their geographical area (Grossenbacher *et al.*, 2015), other causes could also explain the very limited range of *C. grandiflora*: (1) a strong allee effect due to low diversity at self-incompatibility alleles leading to few fertile and viable crosses in marginal populations; (2) hitherto undiscovered ecological factors or human activities altering its natural habitat; or (3) a disruption between plant and pollinators limiting gene flow and species expansion (i.e. a fragmented environment could be responsible, in part, for the success of selfing reproductive systems; Eckert *et al.*, 2009). Finally, the above line of arguments assumes that all *Capsella* species are strict annuals, such that fitness can be compared among species and populations through a single round of reproduction. However, selfing *Capsella* species have a short flowering time period (short life cycle and quick development) and thus it is not excluded that in some parts of the range, especially in warm climates such as in Greece, the selfing species may have a second reproductive period. If so, differences in cumulative fitness over 1 year between selfers and outcrossers could be less than anticipated. In particular, flowering individuals of *C. bursa-pastoris* can be observed almost throughout the whole year (i.e. some individuals flowering in February and other individuals flowering in September; pers. obs. in France and Greece over a period of several years), and we still do not know whether this corresponds to several generations or to sparse germination from a seed bank across a given year. In any case, further studies will be needed to find the causes of the limited geographical range of *C. grandiflora*.

### Implications for the long-term evolution of selfing species

Whatever the underlying causes, the relationship between mating system and competitiveness observed in the present set of experiments suggests new implications for the long-term evolution of selfing species. In particular, it could help resolve the paradox of selfing species that appear to be ecologically and demographically successful in the short term (Grossenbacher *et al.*, 2015), but an evolutionary dead-end in the longer term (Stebbins, 1957; Igic and Busch, 2013; Wright *et al.*, 2013), as illustrated by the *Capsella* genus. Some ecological conditions, such as disturbed, temporary or newly opened habitats, can both favour the evolution of selfing through selection for reproductive assurance, and correspond to weakly competitive environments that would allow their persistence despite poor competitive ability. In the long run, selfing species could be trapped in those habitats where competition is low. An increase in competition, for example during succession, would increase extinction risk by competitive exclusion, especially because the accumulated genetic load would have a stronger demographic impact than under the initially less competitive conditions (Agrawal, 2010; Agrawal and Whitlock, 2012).

A recent meta-analysis suggested that selfing species could experience diminished niche breadth over time despite geographic expansion (Park *et al.*, 2018). The authors suggested that it could be due to the lack of long-term adaptive potential and accumulation of deleterious mutations. Reduced competitive ability could also prevent their establishment in new habitats. The scenario proposed by Park *et al.* (2018) is related to ours where the decrease in available habitats over time would finally reduce geographic range after initial range expansion. This would provide an ecological scenario for the higher extinction rate in selfers underlying the dead-end hypothesis (Goldberg *et al*., 2010; Igic and Busch, 2013) despite initial advantages.

### CONCLUSION

The present study highlighted how profound the impact on the whole life cycle and ecology of a species a shift in mating system can be. Indeed, in both experiments, self-fertilizing individuals had lower performances than cross-fertilizing individuals. In addition, self-fertilizing individuals had lower competitive ability under controlled conditions. The study further demonstrated the importance of taking into account several life history traits at different times across the life cycle and under different conditions to reach a better estimate of fitness. Finally, combining genetic, ecological and demographical approaches has already been advocated to understand the transition from outcrossing to selfing (Cheptou, 2007; Cheptou and Schoen, 2007). We suggest that this should also be a promising approach to a better understanding of the long-term fate of selfing species.

## SUPPLEMENTARY DATA

Supplementary data are available online at https://academic.oup.com/aob and consist of the following. Figure S1: principal component analysis based on life history traits measured to highlight differences relative to the treatment. Figure S2: phenological differences between *Capsella* species. Figure S3: principal component analysis based on life history traits measured to highlight the different genetic clusters within *Capsella bursa-pastoris*. Figure S4: rosette surface without and with competitors in the four species. Table S1: material used in the two experiments. Table S2: germination time under the field experiment. Table S3: analysis of variance for different life history traits under the field experiment. Table S4: analysis of variance for different traits under the controlled experiment.

mcac044_suppl_Supplementary_Figures

mcac044_suppl_Supplementary_Tables
