# Supplementary material for: Competitive ability depends on mating system and ploidy level across Capsella species
Source: Ann Bot. 2022 Mar 25;129(6):697–708. doi: 10.1093/aob/mcac044 (PMC9113120; doi:10.1093/aob/mcac044)

Supplementary Figures

**Figure S1 – Phenological differences between *Capsella* species.** Lifespan (A) and flowering start (B) measured as the number of days after transplant, are represented for *C. bursa-pastoris* (Cbp, in gray), *C. grandiflora* (Cg, in red), *C. orientalis* (Co, in dark blue) and *C. rubella* (Cr, in light blue). The vertical arrows on the x-axis indicate the mean lifespan (A) and the mean flowering start for each species. The stars indicate significant differences between the four species (*p* < 0.05 *, *p* < 0.01 **, *p* < 0.001 ***).

**
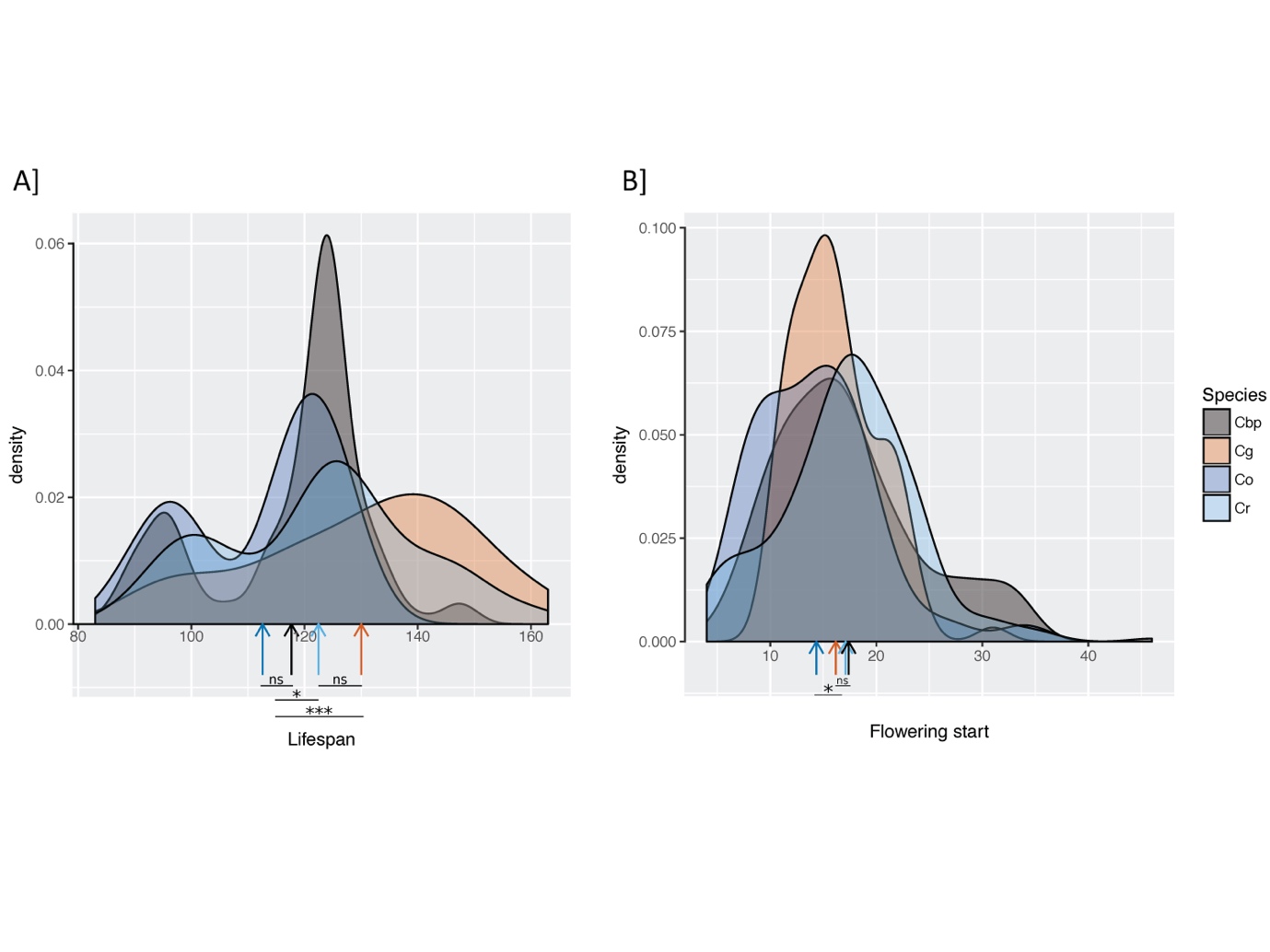
**

**Figure S2 - Principal component analysis (PCA) based on life history traits measured to highlight differences relative to the treatment.** Individuals from weeded (red dots) and unweeded (green triangle) treatments are represented whatever the species. The relative contribution of each variable to the two first principal components is proportional to the length of the arrow (the longer the arrow, the more the variable contributed to the explained variance).

**Figure S3 - Principal component analysis (PCA) based on life history traits measured** to highlight the different genetic clusters within *Capsella bursa pastoris*: European cluster (Cbp_EUR, orange dot), Asian cluster (Cbp_ASI, green square), Middle-Eastern cluster (Cbp_ME, dark blue triangle) and Central Asian cluster (Cbp_CASI, yellow diamond) and showing their relative position to the three other *Capsella* species: *C. grandiflora* (Cg_EUR, orange star), *C. orientalis* (Co, dark blue crosses) and *C. rubella* (Cr, light blue triangle).


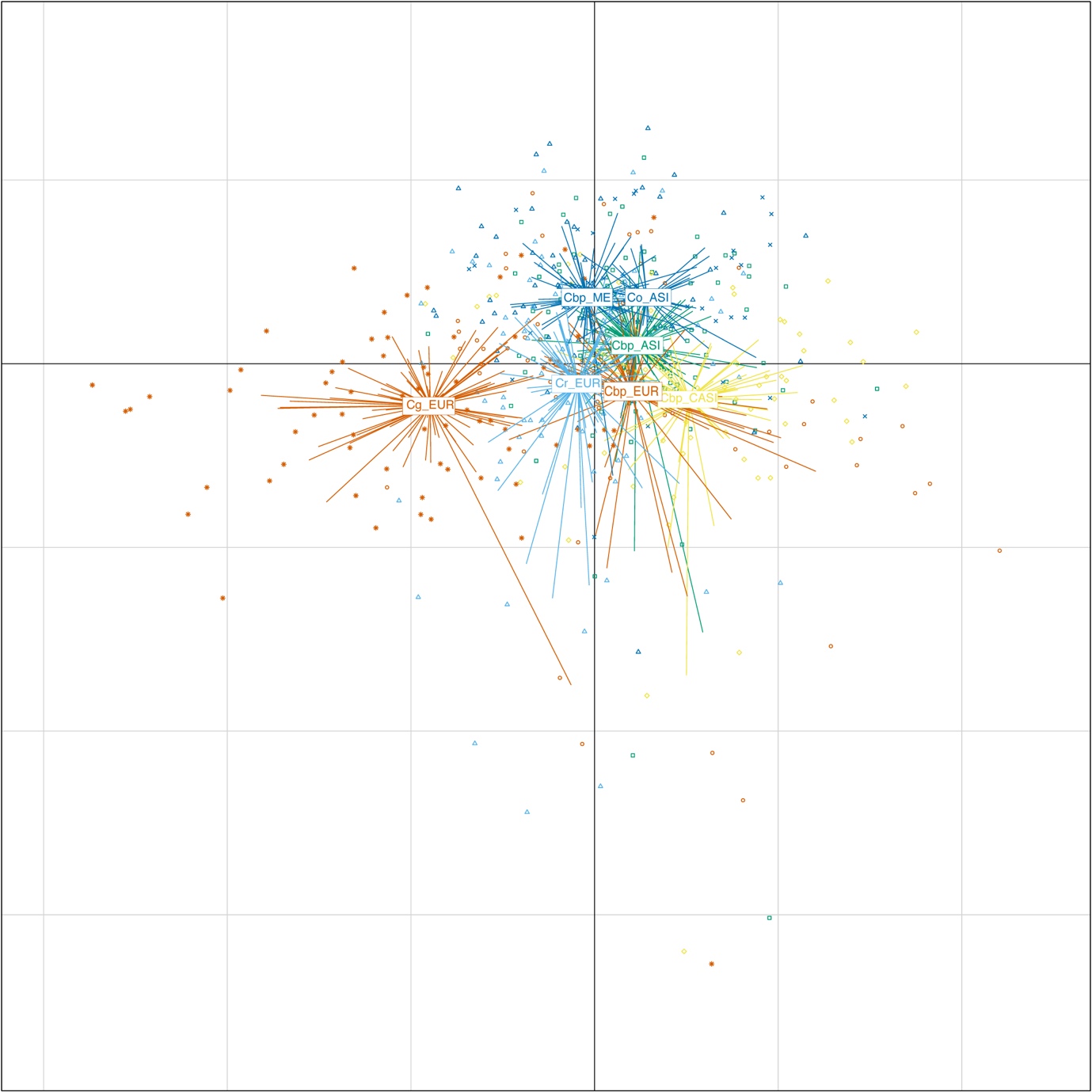


**Figure S4 – Rosette surface** **without and with competitors in the four species.** Each thin line corresponds to one accession (average over the four blocks) and the black lines join the least-square mean estimates (with confidence intervals). For *C. bursa-pastoris*, the color of each line corresponds to the genetic clusters: ASI in green, CASI in yellow, EUR in orange and ME in blue. Ic: competition index. Ic with different letters corresponds to significant treatment x species interactions (see Table S4)


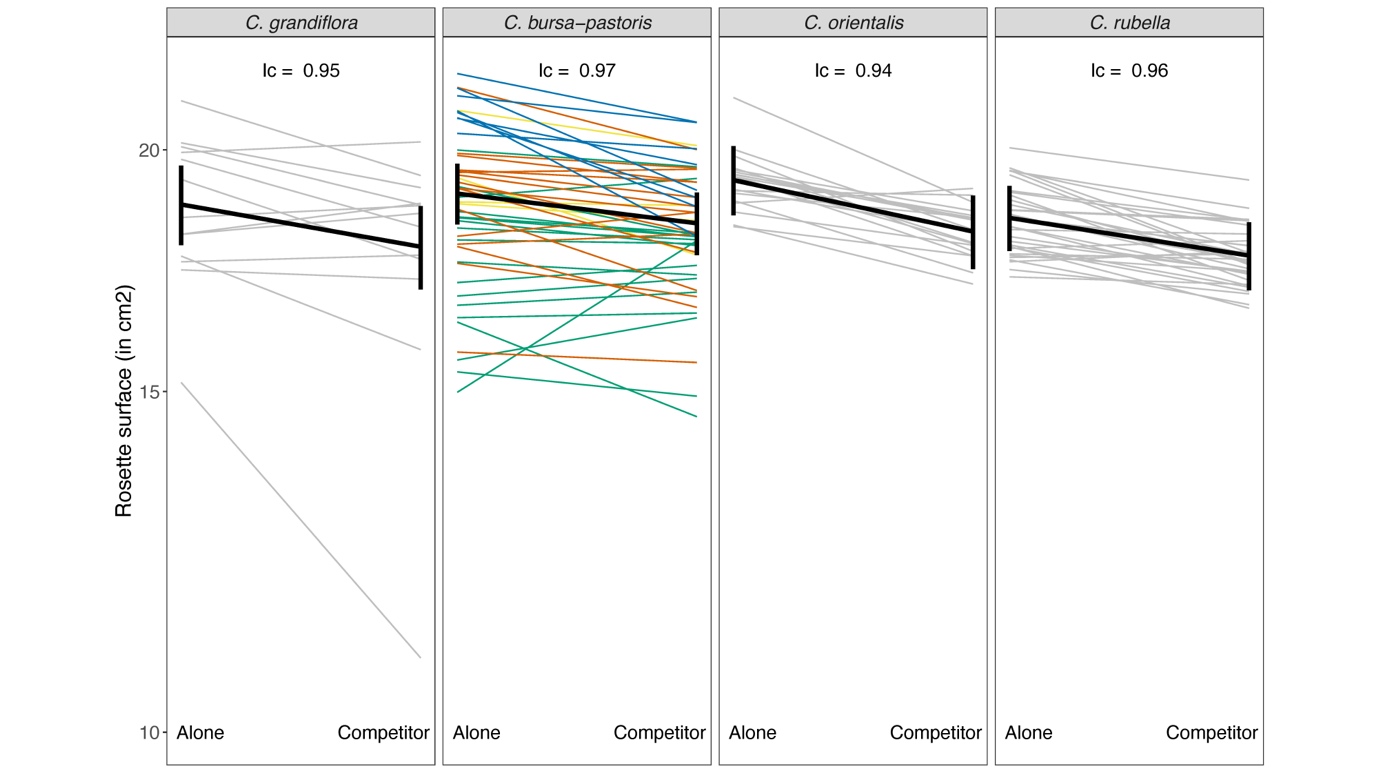

Supplement: mcac044_suppl_Supplementary_Figures [file mcac044_suppl_supplementary_figures.docx]
