# Supplementary material for: Competitive ability depends on mating system and ploidy level across Capsella species
Source: Ann Bot. 2022 Mar 25;129(6):697–708. doi: 10.1093/aob/mcac044 (PMC9113120; doi:10.1093/aob/mcac044)
Supplement: mcac044_suppl_Supplementary_Tables [file mcac044_suppl_supplementary_tables.docx]

**Table S1.** Material used in the two experiments (Controlled environment and Field).

| **Country** | **Area for Cbp** | **Latitude** | **Longitude** | **Accession_number** | **Experiment** |
| --- | --- | --- | --- | --- | --- |
| Greece |  | 39,8 | 21,27 | 28 | Controlled |
| Albania |  | 39,92 | 20,21 | 1 | Controlled |
| Albania |  | 39,91 | 20,01 | 25 | Controlled |
| Albania |  | 40,49 | 19,83 | 28 | Controlled |
| Greece |  | 39,8 | 21,27 | 16 | Controlled |
| Greece |  | 39,8 | 21,27 | 86,1 | Controlled |
| Greece |  | 39,8 | 21,27 | 86,9 | Controlled |
| Greece |  | 39,8 | 21,27 | 87,26 | Controlled |
| Greece |  | 39,8 | 21,27 | 87,32 | Controlled |
| Greece |  | 39,8 | 21,27 | 88,5 | Controlled |
| Greece |  | 39,8 | 21,27 | 96,6 | Controlled |
| Greece |  | 39,8 | 21,27 | 96,7 | Controlled |
| Greece |  | 39,8 | 21,27 | 99,22 | Controlled |
| Greece |  | 39,66 | 20,85 | Cg1.1 | Field |
| Greece |  | 39,66 | 20,85 | Cg1.37 | Field |
| Greece |  | 39,88 | 20,71 | Cg2.19 | Field |
| Greece |  | 39,88 | 20,71 | Cg2.2 | Field |
| Greece |  | 39,90 | 20,71 | Cg5.27 | Field |
| Greece |  | 39,96 | 20,71 | Cg57 | Field |
| Greece |  | 39,30 | 20,98 | Cg26 | Field |
| Greece |  | 39,41 | 20,83 | Cg3 | Field |
| Greece |  | 39,41 | 20,83 | Cg9.1 | Field |
| Greece |  | 39,96 | 20,71 | Cg9.2 | Field |
| Italy |  | 39,36 | 16,23 | 75,13 | Controlled |
| Italy |  | 39,36 | 16,23 | 79,17 | Controlled |
| Spain |  | 43,09 | 6,25 | 82,16 | Controlled |
| Italy |  | 40,94 | 15,2 | 73,6 | Controlled |
| Greece |  | 38,09 | 22,4 | 74,9 | Controlled |
| Greece |  | 37,69 | 21,63 | 84,2 | Controlled |
| Greece |  | 37,73 | 21,69 | 80,15 | Controlled |
| Greece |  | 39,56 | 20,92 | 80,2 | Controlled |
| Greece |  | 39,56 | 20,92 | 80,13 | Controlled |
| France |  | 45,81 | 5,77 | 83,2 | Controlled |
| Greece |  | 39,56 | 20,92 | 82,17 | Controlled |
| France |  | 43,39 | 0,05 | VIC-FR7-3 | Controlled |
| France |  | 43,39 | 0,05 | VIC-FR7-4 | Controlled |
| France |  | 43,33 | 0,1 | TOS-FR8-3 | Controlled |
| France |  | 43,33 | 0,1 | TOS-FR8-4 | Controlled |
| France |  | 45 | 2,5 | VIB-FR10-1 | Controlled |
| France |  | 45 | 2,5 | VIB-FR10-2 | Controlled |
| France |  | 45 | 2,5 | VIB-FR10-3 | Controlled |
| France |  | 42,67 | 0,66 | AUZ-FR11-2 | Controlled |
| France |  | 42,67 | 0,66 | AUZ-FR11-5 | Controlled |
| France |  | 43,33 | 4,85 | MOS-FR12-3 | Controlled |
| Greece |  | 40,65 | 22,9 | THE-GR2-1 | Controlled |
| Greece |  | 40,65 | 22,9 | THE-GR2-4 | Controlled |
| Palestine |  | 32,23 | 35,26 | NAB-PAL1-6 | Controlled |
| Palestine |  | 32,23 | 35,26 | NAB-PAL1-7 | Controlled |
| Palestine |  | 32,23 | 35,26 | NAB-PAL3-4 | Controlled |
| Palestine |  | 32,23 | 35,26 | NAB-PAL3-9 | Controlled |
| France |  | 43,39 | 0,05 | VIC-FR7-2 | Controlled & Field |
| France |  | 43,33 | 0,1 | TOS-FR8-6 | Controlled & Field |
| Greece |  | 40,65 | 22,9 | THE-GR2-5 | Controlled & Field |
| Palestine |  | 32,23 | 35,26 | NAB-PAL3-5 | Controlled & Field |
| Greece |  | 41,69 | 26,38 | Cr1.1 | Field |
| Greece |  | 41,69 | 26,38 | Cr1.2 | Field |
| Greece |  | 41,69 | 26,38 | Cr1.3 | Field |
| Greece |  | 41,69 | 26,38 | Cr1.4 | Field |
| Greece |  | 37,89 | 22,73 | Cr73.1 #4 | Field |
| Greece |  | 37,74 | 21,69 | Cr 79.9 #4 | Field |
| Russia |  | 53,3 | 60,1 | par9 | Controlled |
| Russia |  | 51,29 | 58,18 | 4 | Controlled |
| Russia |  | 51,29 | 58,18 | 6 | Controlled |
| Russia |  | 51,29 | 58,18 | 5 | Controlled |
| Russia |  | 53,3 | 60,1 | 2 | Controlled |
| Russia |  | 53,3 | 60,1 | 3 | Controlled |
| Russia |  | 53,3 | 60,1 | 4 | Controlled |
| Russia |  | 53,3 | 60,1 | 5 | Controlled |
| Russia |  | 54,32 | 62,68 | 6 | Controlled |
| Russia |  | 54,32 | 62,68 | 7 | Controlled |
| China |  | 46,7 | 90,83 | 1 | Controlled |
| China |  | 46,7 | 90,83 | 2 | Controlled |
| China |  | 46,7 | 90,83 | 4 | Controlled |
| China |  | 46,7 | 90,83 | 10 | Controlled |
| Russia |  | 55,11 | 61,39 | 9 | Controlled |
| Russia |  | 51,29 | 58,18 | 2 | Controlled & Field |
| Russia |  | 54,32 | 62,68 | 3 | Controlled & Field |
| Russia |  | 54,32 | 62,68 | 4 | Controlled & Field |
| Russia |  | 55,11 | 61,39 | 4 | Controlled & Field |
| Russia |  | 55,11 | 61,39 | 7 | Controlled & Field |
| China |  | 46,70 | 90,83 | QH-CHIN-1 | Field |
| France | Europe | 48,08 | 7,37 | FR50 | Controlled |
| France | Europe | 44,51 | -1,21 | STJ2 | Controlled |
| UK | Europe | 56,2 | -2,47 | STA4 | Controlled |
| Suède | Europe | 62,64 | 17,94 | SE14 | Controlled |
| Suède | Europe | 59,87 | 17,63 | SE42 | Controlled |
| Italy | Europe | 41,17 | 13,57 | 22_17 | Controlled |
| Italy | Europe | 37,09 | 15,2 | 39_12 | Controlled |
| Espagne | Europe | 42,24 | 1,5 | 53_7 | Controlled |
| France | Europe | 44,33 | -0,21 | 6_19 | Controlled |
| Czech Republic | Europe | 49,2 | 16,63 | CZ96 | Controlled |
| Greece | Europe | 36,17 | 28 | GR90 | Controlled |
| Bosnia_Hercegovina | Europe | 43,51 | 18,25 | TON_4 | Controlled |
| Russia | Europe | 50,55 | 128,28 | BEL_6 | Controlled |
| Russia | Europe | 51,23 | 109,51 | BAD_1 | Controlled |
| Russia | Europe | 52,16 | 104,18 | IRRU3 | Controlled |
| Russia | Europe | 48,28 | 135,05 | KAB_5 | Controlled |
| Russia | Europe | 53,44 | 119,45 | MOG_1 | Controlled |
| Russia | Europe | 51,39 | 39,11 | VORU1 | Controlled |
| Russia | Europe | 49 | 131,03 | OBL_4 | Controlled |
| Jordan | Middle-East | 31,97 | 35,98 | JO59 | Controlled |
| Israel | Middle-East | 32,6 | 35,13 | MAE_4 | Controlled |
| Algeria | Middle-East | 36,77 | 5,08 | AL88 | Controlled |
| Syria | Middle-East | 33,5 | 36,3 | SY64 | Controlled |
| Syria | Middle-East | 35,33 | 40,15 | SY67 | Controlled |
| Syria | Middle-East | 36,2 | 37,17 | SY68 | Controlled |
| China | Asia | 38,56 | 121,35 | DL_174 | Controlled |
| China | Asia | 26,53 | 112,33 | HY85 | Controlled |
| China | Asia | 30,16 | 120,13 | HJC419 | Controlled |
| China | Asia | 32,03 | 118,46 | NJ219 | Controlled |
| China | Asia | 37,55 | 112,32 | TY118 | Controlled |
| China | Asia | 26,37 | 106,43 | GY_35 | Controlled |
| China | Asia | 30,31 | 117,05 | AQ_413 | Controlled |
| China | Asia | 39,55 | 116,22 | BJA162 | Controlled |
| China | Asia | 39,56 | 116,2 | BJB_240 | Controlled |
| China | Asia | 28,12 | 113,05 | CSH_8 | Controlled |
| China | Asia | 36,36 | 114,29 | HD_70 | Controlled |
| China | Asia | 31,51 | 117,17 | HF_254 | Controlled |
| China | Asia | 30,16 | 120,13 | HJC_418 | Controlled |
| China | Asia | 45,45 | 126,37 | HRB_137 | Controlled |
| China | Asia | 30,12 | 115,06 | HSH_298 | Controlled |
| China | Asia | 29,39 | 115,59 | JJ_392 | Controlled |
| China | Asia | 25,05 | 102,41 | KMA_373 | Controlled |
| China | Asia | 25,06 | 102,41 | KMB_215 | Controlled |
| China | Asia | 27,37 | 113,5 | PX_269 | Controlled |
| China | Asia | 35,36 | 116,58 | QF_341 | Controlled |
| China | Asia | 39,38 | 118,11 | TSH_191 | Controlled |
| China | Asia | 30,37 | 114,16 | WH_46 | Controlled |
| China | Asia | 34,16 | 108,52 | XA_110 | Controlled |
| China | Asia | 36,37 | 101,46 | XN_442 | Controlled |
| China | Asia | 32,09 | 114,04 | XY_18 | Controlled |
| China | Asia | 27,47 | 114,23 | YC_312 | Controlled |
| China | Asia | 28,36 | 112,18 | YY_381 | Controlled |
| China | Asia | 34,45 | 113,4 | ZZH_279 | Controlled |
| Russia | Central_asia | 51,53 | 58,85 | DUB-RUS9 | Controlled |
| Kazakhstan | Central_asia | 47 | 83,01 | TACH-CHIN14 | Controlled |
| Kirghizistan | Central_asia | 40 | 72 | KIRG-3-14 | Controlled |
| Kirghizistan | Central_asia | 40 | 73 | KIRG-7 | Controlled |
| China | Asia | 23,97 | 120,95 | PL | Contolled & Field |
| China | Asia | 33,57 | 107,45 | TSB | Contolled & Field |
| Suède | Europe | 56,15 | 13,77 | SE33 | Controlled & Field |
| Russia | Europe | 43,13 | 131,4 | VLA_3 | Controlled & Field |
| Turkey | Middle-East | 41,02 | 28,97 | TR73 | Controlled & Field |
| Jordan | Middle-East | 31,97 | 35,98 | JO56 | Controlled & Field |
| Algeria | Middle-East | 35,45 | 7,96 | AL87 | Controlled & Field |
| China | Asia | 30,2 | 112,06 | JZH152 | Controlled & Field |
| China | Central_asia | 47,07 | 83,01 | TACH-CHIN-6 | Field |
| Kirghizistan | Central_asia | 39,79 | 72,18 | KIRG-5-12 | Field |
| China | Asia | 36,37 | 101,46 | XN444 | Field |
| China | Europe | 45,45 | 126,37 | HRB132* | Field |
| China | Asia | 30,16 | 120,13 | HJC421 | Field |
| Russia | Europe | 52,24 | 104,27 | IRRU2 | Field |
| US | Middle-East | 31,55 | -97,15 | WAC5 | Field |
| Russia | Europe | 59,93 | 30,33 | PETER-RUS-10 | Field |
| China | Central_asia | 48,33 | 87,12 | BERG-CHIN-3 | Field |

* sampled in China but clustering with the European ones

**Table S2.** Germination time under the field experiment

| **Species** | **Accession ID** | **Origin** | **Germination date (> 10 seeds)** | **number of days to reach at least 10 seeds)** |
| --- | --- | --- | --- | --- |
| *C. grandiflora* | Cg1 | EUR | 2018-01-28 | 16 |
|  | Cg14 | EUR | 2018-01-28 | 16 |
|  | Cg16 | EUR | 2018-01-28 | 16 |
|  | Cg17 | EUR | 2018-01-24 | 12 |
|  | Cg27 | EUR | 2018-01-25 | 13 |
|  | Cg33 | EUR | 2018-02-20 | 39 |
|  | Cg35 | EUR | 2018-01-25 | 13 |
|  | Cg36 | EUR | 2018-01-30 | 18 |
|  | Cg37 | EUR | 2018-01-23 | 11 |
|  | Cg40 | EUR | 2018-01-25 | 13 |
| *C. rubella* | Cr1 | EUR | 2018-01-23 | 11 |
|  | Cr2 | EUR | 2018-01-23 | 11 |
|  | Cr3 | EUR | 2018-01-24 | 12 |
|  | Cr4 | EUR | 2018-01-23 | 11 |
|  | Cr8 | EUR | 2018-01-22 | 10 |
|  | Cr10 | EUR | 2018-01-22 | 10 |
|  | Cr11 | EUR | 2018-01-30 | 18 |
|  | Cr12 | EUR | 2018-01-30 | 18 |
|  | Cr14 | EUR | 2018-01-23 | 11 |
|  | Cr15 | ME | 2018-01-29 | 17 |
| *C. orientalis* | Co1 | ASI | 2018-01-23 | 11 |
|  | Co5 | CASI | 2018-01-23 | 11 |
|  | Co6 | CASI | 2018-01-23 | 11 |
|  | Co7 | CASI | 2018-01-23 | 11 |
|  | Co9 | CASI | 2018-01-23 | 11 |
|  | Co10 | CASI | 2018-01-23 | 11 |
| *C. bursa pastoris* | Cbp2 | EUR | 2018-02-25 | 44 |
|  | Cbp8 | EUR | 2018-01-24 | 12 |
|  | Cbp9 | EUR | 2018-01-23 | 11 |
|  | Cbp10 | EUR | 2018-01-23 | 11 |
|  | Cbp11 | ME | 2018-01-22 | 10 |
|  | Cbp12 | ME | 2018-01-22 | 10 |
|  | Cbp13 | ME | 2018-01-22 | 10 |
|  | Cbp14 | ME | 2018-01-22 | 10 |
|  | Cbp15 | ASI | 2018-02-20 | 39 |
|  | Cbp18 | ASI | 2018-02-20 | 39 |
|  | Cbp20 | ASI | 2018-01-24 | 12 |
|  | Cbp21 | ASI | 2018-02-26 | 45 |
|  | Cbp22 | ASI | 2018-02-20 | 39 |
|  | Cbp23 | ASI | 2018-02-15 | 34 |
|  | Cbp27 | CASI | 2018-01-24 | 12 |
|  | Cbp29 | EUR | 2018-02-20 | 39 |
|  | Cbp30 | CASI | 2018-01-23 | 11 |
|  | Cbp31 | CASI | 2018-01-23 | 11 |
|  |  |  |  |  |

**Table S3.** Analysis of variance for different life history traits under the field experiment. Significant effects are highlighted. RS: Reproductive system, SP: Species, PL: Ploidy, GC: Genetic cluster. See Text for additional details.

|  |  |  | **Number of fruits** | | **Rosette size** | | **Lifespan** | | **Flowering start** | | **Germination rate** | | **Fitness Index** | |
| --- | --- | --- | --- | --- | --- | --- | --- | --- | --- | --- | --- | --- | --- | --- |
|  |  | df | LR | P-value | LR | P-value | LR | P-value | F-value | P-value | LR | P-value | LR | P-value |
| **Models using 4 species** | damage | 1 | 0,25 | 0,62 | 0,19 | 0,66 | 0,26 | 0,61 | 0,02 | 0,89 | - | - | 1,91 | 0,17 |
|  | Env. | 1 | 0,44 | 0,51 | 0,55 | 0,46 | 0,01 | 0,93 | 0,01 | 0,91 | **16,99** | **<0.001** | 1,12 | 0,29 |
|  | RS | 1 | **45,9** | **<0.001** | **5,92** | **<0.01** | **14,44** | **<0.001** | 2 | 0,16 | **18,66** | **<0.001** | **21,29** | **<0.001** |
|  | Env x RS | 1 | 0,27 | 0,61 | 1,42 | 0,06 | 0,14 | 0,71 | 0 | 0,96 | 2,75 | 0,09 | 0,29 | 0,59 |
|  | damage | 1 | 0,2 | 0,66 | 0,19 | 0,66 | 0,23 | 0,63 | 0,09 | 0,76 | - | - | 1,63 | 0,2 |
|  | Env. | 1 | 0,22 | 0,64 | 0,55 | 0,46 | 0,97 | 0,32 | 0,34 | 0,56 | **7,13** | **<0.01** | 0,03 | 0,86 |
|  | SP | 3 | **48** | **<0.001** | **16,85** | **<0.001** | **27,39** | **<0.001** | **25,8** | **<0.001** | **115,78** | **<0.001** | **35,18** | **<0.001** |
|  | Env x SP | 3 | 2,65 | 0,45 | 1,31 | 0,73 | 0,54 | 0,91 | 0,49 | 0,48 | **13,14** | **<0.01** | 4,47 | 0,22 |
| **Models on the 3 selfing species** | damage | 1 | 0,31 | 0,58 | 0,06 | 0,79 | 0,65 | 0,42 | 0,07 | 0,79 | - | - | 2,07 | 0,15 |
|  | Env. | 1 | 0,13 | 0,72 | 0,58 | 0,44 | 0,01 | 0,99 | 0,03 | 0,86 | **24,93** | **<0.001** | **4,74** | **<0.05** |
|  | PL | 1 | 0,31 | 0,58 | **5,88** | **< 0.01** | 1,66 | 0,2 | **4,18** | **<0.05** | **132,28** | **<0.001** | **17,46** | **<0.001** |
|  | Env x PL | 1 | 0,17 | 0,68 | 1,15 | 0,28 | 0,37 | 0,54 | 0,17 | 0,68 | **7,08** | **<0.01** | 3,6 | 0,06 |
|  | damage | 1 | 0,3 | 0,58 | 0,02 | 0,89 | 0,51 | 0,48 | 0,01 | 0,91 | - | - | 1,94 | 0,16 |
|  | Env. | 1 | 0,05 | 0,83 | 0,6 | 0,44 | 1,02 | 0,31 | 0,32 | 0,57 | **7,32** | **<0.01** | 0,02 | 0,9 |
|  | SP | 2 | 1,02 | 0,6 | **9,81** | **<0.01** | **12,24** | **<0.01** | **31,24** | **<0.001** | **130,73** | **<0.001** | **17,09** | **<0.001** |
|  | Env x SP | 2 | 1,12 | 0,57 | 1,44 | 0,49 | 0,42 | 0,81 | 2,2 | 0,33 | **7,01** | **<0.01** | 3,8 | 0,15 |
| **Model intra-species (*C. bursa-pastoris)*** | damage | 1 | 0,97 | 0,32 | 0,98 | 0,32 | 0,33 | 0,56 | 2,33 | 0,13 | - | - | 0,57 | 0,45 |
|  | Env. | 1 | 0,03 | 0,85 | 0,36 | 0,55 | 0,34 | 0,56 | 3,32 | 0,07 | 0,12 | 0,73 | 0,01 | 0,97 |
|  | GC | 3 | **20,22** | **<0.01** | 3,42 | 0,33 | **8,81** | **<0.05** | **9,3** | **<0.05** | **51,74** | **<0.001** | **9,53** | **<0.05** |
|  | Env x GC | 3 | 3,89 | 0,27 | 2,99 | 0,39 | 0,82 | 0,84 | 7,75 | 0,06 | **23,71** | **<0.001** | 2,06 | 0,56 |
| df, degrees of freedom; LR, Likelihood ratio. The significant terms are hiighlighted in bold | | | | | | |  |  |  |  |  |  |  |  |

**Table S4** Analysis of variance for different traits under the controlled experiment. SP: Species, GC: Genetic cluster, Treatment: weeded or unweeded.

|  |  |  | **Diameter t1** | | **Diameter t2** | | **Growth rate** | | **Flowering rate** | | **Number of flower** | |
| --- | --- | --- | --- | --- | --- | --- | --- | --- | --- | --- | --- | --- |
|  |  | df | Chisq | P-value | Chisq | P-value | Chisq | P-value | Chisq | P-value | Chisq | P-value |
| **Models using 4 species** | SP | 3 | 4,31 | 0,23 | 6,25 | 0,1 | 1,07 | 0,78 | 5,9 | 0,12 | **172,28** | **<0.001** |
|  | treatment | 1 | **12,81** | **<0.001** | **17,77** | **<0.001** | 0 | 0,99 | 1,03 | 0,31 | **55,99** | **<0.001** |
|  | SP x treatment | 3 | 5,54 | 0,14 | 5,86 | 0,12 | 3,99 | 0,26 | 1,09 | 0,78 | **95,49** | **<0.001** |
| **Model intra-species  (*C. bursa-pastoris)*** | GC | 3 | **25,72** | **<0.001** | **54,32** | **<0.001** | 3,65 | 0,3 | 1,56 | 0,67 | 2,37 | 0,5 |
|  | treatment | 1 | **14,64** | **<0.001** | **42,58** | **<0.001** | **4,22** | **<0.05** | 0 | 0,99 | **36,51** | **<0.001** |
|  | GC x treatment | 3 | 6,99 | 0,07 | **23,3** | **<0.001** | 3,36 | 0,34 | 1,71 | 0,63 | **40,81** | **<0.001** |
